# The influence of context and process when implementing e-health

**DOI:** 10.1186/1472-6947-9-9

**Published:** 2009-01-30

**Authors:** David Boddy, Gerry King, Julia S Clark, David Heaney, Frances Mair

**Affiliations:** 1 Department of Management, University of Glasgow, Glasgow, Scotland, UK; 2 Centre for Rural Health, University of Aberdeen, Centre for Health Sciences, Inverness, Scotland, UK; 3 Department of General Practice and Primary Care, University of Glasgow, Glasgow, Scotland, UK

## Abstract

**Background:**

Investing in computer-based information systems is notoriously risky, since many systems fail to become routinely used as part of everyday working practices, yet there is clear evidence about the management practices which improve the acceptance and integration of such systems. Our aim in this study was to identify to what extent these generic management practices are evident in e-health projects, and to use that knowledge to develop a theoretical model of e-health implementation. This will support the implementation of appropriate e-health systems.

**Methods:**

This study consisted of qualitative semi-structured interviews with managers and health professionals in Scotland, UK. We contacted the Scottish Ethics Committee, who advised that formal application to that body was not necessary for this study. The interview guide aimed to identify the issues which respondents believed had affected the successful implementation of e-health projects. We drew on our research into information systems in other sectors to identify likely themes and questions, which we piloted and revised. Eighteen respondents with experience of e-health projects agreed to be interviewed. These were recorded, transcribed, coded, and then analysed with 'Nvivo' data analysis software.

**Results:**

Respondents identified factors in the context of e-health projects which had affected implementation, including clarity of the strategy; supportive structures and cultures; effects on working processes; and how staff perceived the change. The results also identified useful implementation practices such as balancing planning with adaptability; managing participation; and using power effectively.

**Conclusion:**

The interviews confirmed that the contextual factors that affect implementation of information systems in general also affect implementation of e-health projects. As expected, these take place in an evolving context of strategies, structures, cultures, working processes and people. Respondents also confirmed that those managing such projects seek to change these contexts through observable implementation processes of planning, adaptation, participation and using power. This study confirms that research to support the delivery of appropriate e-health projects can usefully draw on the experience of information systems in other sectors.

## Background

Spending money on computer-based information systems is a notoriously risky enterprise, as the costs and disruption are usually much easier to demonstrate than the benefits, thus creating challenges for those promoting such systems. It is equally clear from research in many sectors of the economy [1-3] that the acceptance and use of computer-based information systems depends on those responsible ensuring that changes in organisation complement changes in technology. A multi-disciplinary team of researchers from Glasgow, Dundee and Aberdeen Universities has conducted a study, referred to as the HAVEN project, examining the extent to which these factors influence successful implementation and integration of e-health technologies. In the is study we defined 'e-health' broadly, as the application of information and communication technologies (ICT) across the whole range of functions which may affect the health of citizens and patients.

While health care has unique features, some generic management processes may be common to all organisations. If so, this would inform the development of an empirically-based theoretical model of e-health implementation, which in turn could support the delivery of appropriate e-health systems in Scotland and elsewhere. The HAVEN study included: a scoping exercise on e-health research in Scotland [4]; a systematic review of e-Health research; and citizens' juries to identify beliefs about research needs. It also included an interview programme which is the subject of this paper. While our focus is on e-Health in Scotland, the themes are likely to be relevant in other health care systems.

**Figure 1:**
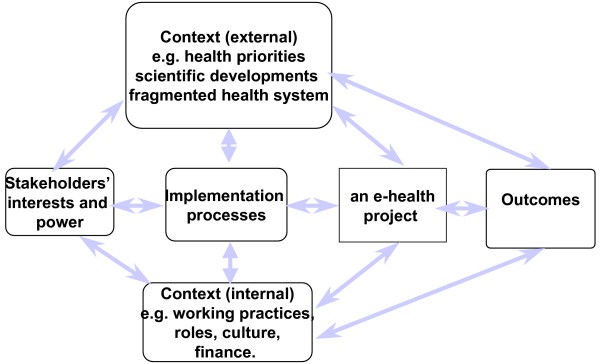
**An interaction model of e-health implementation**.

Figure 1 integrates the conclusions of research on computer-based information systems in many sectors of the economy [1,3], and in the present context aims to guide those responsible (at any level of a health care system) for designing and implementing e-health projects. It shows that outcomes depend on the actions of stakeholders with an interest in the project [5,6]. These actions constitute an implementation process (whether formally established, haphazard, or perhaps both) which takes place within a context [7] with external and internal dimensions. The external context includes social, political and other features of the world beyond, while the internal refers to features of the organisation itself within which e-health projects evolve. Stakeholders interact [1] with each other, and with features of the wider context, as they try to implement or block an e-health project. For example one line of research has focused on the relation between information systems and the wider strategy of the host unit [8], while others have focused on the links between a new system and established routines [9,10]. Some researchers [10-12] have examined the effects of structures and cultures on the acceptance of new systems, while others [13] examine the factors influencing whether people accept and use them.

Some have studied one or more components of the model – for example, in relation to the external context, [14] showing how the contentious political and social contexts have affected the progress of the UK's National Programme for Information Technology. Studies of the internal context include [15] work on the relation between e-health systems and strategy, [16] on the structural tensions which arise when e-health systems are designed nationally but delivered locally by relatively autonomous institutions, and [17] on the influence of professional cultures on the acceptance of new health technologies. Our study builds on this work by incorporating it and other work within Figure 1, and using that integrated model to guide our study of beliefs about why some e-health projects succeed and others fail. That in turn would indicate the agendas which those responsible need to manage to promote successful implementation and integration of e-health services.

## Methods

Figure 1 together with the normalization process model [18,19] informed the design of the interview schedule. This began with questions about the interviewee's role in the service, and the e-health projects of which they had experience. It then moved to each of the headings in the model, asking respondents to comment on the extent to which utilisation had been affected by, for example, aspects of the external or internal contexts, with brief prompts being available to encourage commentary or to clarify the question. The draft schedule was piloted with three respondents, leading to revisions.

Eighteen respondents were purposively recruited for interview, including policymakers within the Scottish Government Health Department, senior personnel within health boards; clinicians with experience of e-health applications; and suppliers. They had experience of e-health systems across 4 domains of e-health: management, communication, computerized decision support, and information systems [20] but in this preliminary study we did not analyse the replies against these categories. One of the two researchers interviewed each respondent in person for about one hour. The interviews were recorded, transcribed by secretarial staff and corrected by the researchers.

Analysis began by developing the coding schedule, using the interview schedule to prepare a draft. Five members of the team used this to code two transcripts independently. They then met to check the consistency of their respective interpretations of the transcripts and codes. This led to a final coding schedule, against which two further transcripts were coded independently by three members of the team. At this stage coding was identified as being consistent but further codes were added to include issues of process, which were tested on a further transcript. The codes were then entered into the qualitative data analysis program 'Nvivo', which was used to organise the data and structure the analysis. The researchers then coded the transcripts, identified the main themes; and traced possible relationships. In practice analysis had begun during data collection, as notes made after each interview offered early insights and indicated points to explore in subsequent interviews.

## Results

The interviews identified features of the external context which influence the management of e-health projects, such as an increasingly IT-literate population which is critical of inefficient paper systems; differences between professions about the security of patient data; and ambiguity about the cost-effectiveness of e-health. A feature of particular relevance to the future of e-health in Scotland is that while the health care system is funded nationally, the geographical health boards have considerable autonomy over expenditure. Some have spent heavily on e-health, while others have given it a lower priority: moreover, the enthusiasts often develop independent systems which are incompatible with those of other boards.

Health board autonomy is clearly a major factor in the external context affecting those developing e-health systems in Scotland, but in this paper we focus mainly (though not exclusively) on evidence about the internal context and the processes of implementation. These represent the immediate setting of an e-health project, reflect earlier local decisions, and so are probably within the authority of those developing and implementing e-health projects. Factors in the internal context which arose most frequently were those of strategy, structure, culture, working processes and people: process issues were those of planning and flexibility, participation, and power. The following pages illustrate each theme.

## Internal context

### Strategy

This theme usually links the external and internal contexts, and these interviews were consistent with that. Several advocated the benefits of developing a national strategy on e-health, which some suggested was lacking. Respondent (01) noted that while having a clear national strategy would help set spending priorities, local decisions need to support these. They cited a health board which had decided to use a nationally-approved e-health application, but had failed to allocate funds to purchase successive local upgrades, so frustrating clinicians' expectations. Another interviewee (11) suggested that strategy should reflect clinical priorities:

There's a big demand for lab results to pushed into the GP system and we're still waiting for that to happen, and that's one of the many things that there's been quite slow progress with. [It's] very high on the wish list of most GPs to get lab results coming into their system automatically.

Respondent (05) commented that as well as giving a sense of purpose to investment decisions, a strategy should also indicate which applications would be provided and funded nationally, and which left to local decisions:

There's some disjointedness in the system which creates a lot of friction and slower progress [than is desirable].

Respondent (07) illustrated this by referring to a system that had been approved for use nationally, but which health boards had been slow to implement. During that time a rival package had appeared, which many people had started to use in preference to the one which NHS Scotland was promoting.

A recurring theme was the benefits that would follow if e-health strategy was embedded as part of health strategy, rather than being seen as an optional extra. This would help to clarify how investing in e-health systems contributed to service provision, which could then be compared with other investments. For example respondent (13) explained how e-health systems in their board were improving health system performance, as measured by waiting times and other indicators:

Many health boards are just not aware of what is happening, and make decisions on aggregate and historical data...I think we're beginning to see the benefits of having real time data on which to make decisions.

Respondent (04) recalled how they had won the support of the chief executive, who made e-health an essential part of the working practices of senior managers; their key performance indicators now included measures reflecting the use of e-health systems.

### Structure

This relates to the way the tasks required to deliver a service are divided and coordinated, and to the operating mechanisms (like appraisals) which encourage specified behaviour. A common theme was that health board autonomy led to different strategies towards e-health, and to incompatible local systems. This was partly due to different technologies, but also because people adapt working practices to fit the technology: both forces inhibit acceptance of national systems. As (01) said:

We've sorted out [a national solution], but then our lab system won't feed the correct data. A lot of it's to do with the fact that Scotland has multiple systems for doing exactly the same thing, which is ludicrous. Some boards have put money into e-health and others haven't.

Other respondents mentioned the challenge of imposing a national policy when health boards are able to take an independent line, rather than follow national guidance – several implied that NHS Scotland needed to encourage boards to adopt national e-health systems if they were to realize economies of scale. A board's allocation to support e-health also affects how well they use national systems:

A generic clinical system in one department went live last month, but we haven't got a huge amount of resource locally within e-health to back it up while clinicians learn to use it.

Respondent (04) gave an example how incentives had influenced behaviour:

There was a real improvement in consultants' attitudes to e-health when the consultants' contract was implemented, and we now have to do appraisals with consultants. That involves workload analysis and they've had to start putting things in themselves and making sure the data is right.

Another cited a system which NHS Scotland selected, and for which they offered to pay: if a board chose another system they would have to finance it locally.

### Culture

Culture refers to the beliefs and values that people in an occupation or organisation hold and which, in the present context, affect their responses to e-health. They are likely to accept a system that supports their values, and oppose one which challenges them. Respondent (03):

We're a fairly forward-looking practice and I like to think that we're adaptive and move with the times. [But] in some practices you get someone who says "oh no, no" and if you get one person who is not doing it, then it won't work: you all have to do it, and that is why this...project will only work if everyone in Scotland does it.

Professionals in different roles develop distinct views about the role of information, and so of e-health systems. Interviewee (04) suggested that consultants in hospitals.

see themselves as part of a multi-disciplinary team that needs to know about this person, and this means sharing information...The GPs have a different set of feelings, not seeing the patient as a case who comes through the hospital, but as a person with whom they expect to have a relationship over many years. So the way GPs see information is subtly different, and their instinct is to control who could access it.

Such cultural differences influence behaviour. For some it will mean ready acceptance of a system that supports their values and self-image, as expressed above by (01). Others may conclude that a planned system will come between them and their core mission, distracting them from it. The sources of cultural beliefs are relevant to anyone who wishes to influence them. A theory put forward by (14) related medical cultures to training:

Doctors are trained that the buck stops with them; so they say – 'if the buck stops with me, I'll do things my way, the way I feel comfortable with'. So they do things their own way. What becomes very, very difficult is in IT, if you want to put in an IT solution you don't want to put in one for one doctor, and one for another doctor: you're looking for universal common ground, and getting to that point is very difficult.

Respondent (05) referred to the emphasis in training to body language and demeanor – so that if technology then appears to place a barrier in the way of such observations, staff may doubt their ability to make an adequate judgment.

### Working processes

These are the activities that people and technologies perform on materials and information, and e-health systems often enable significant changes. For example, one widely adopted system reported by (03) led to changes in the health service's regional offices and in the GP practices:

The regional offices had to completely change the way that they worked because they now had to be able to handle electronic records coming back to them and re-routing them. So it was a completely new way of working, and if a practice wasn't able cope, or if the patient was moving to England, then in those cases they had to be prepared to print them off centrally. So there were a lot of changes, and it took a lot of willingness on everyone's part to make it work. They were asked to completely change their way of working, which they did.

There were also changes in the GP practices, where instead of receiving and filing paper, administrators now use a keyboard to update patients' records. This required instruction, so:

Somebody on the project team prepared a lot of information on what to do. They prepared various checklists which staff in the practices have to go through... and there were several workshops and meetings where the pilot projects fed back and said 'this is good but you can do better, this is how we would refine the process'. So the next practices didn't have to go through all the teething troubles. The project management was probably the biggest bit.

In this practice it also changed the way GPs worked:

Instead of having a practice meeting where we sit around and discuss all the letters and read them altogether, we now go to the office and read them on the machine. It's not a team activity any more. I don't particularly miss the team bit – I actually find it slightly easier now.

One effect of implementing an e-health system is to draw attention to current working processes, which become more visible and open to scrutiny. As (02) noted:

After implementation it was apparent that our understanding of the processes of care, the way the hospital operated, wasn't quite as clear as it should have been. That was partly because we hadn't found out about it, partly because people would tell you how they did something in theory, but didn't tell you how they actually did it in practice. The user requirement hadn't been understood.

### People

This heading includes staff of all kinds delivering care as well as patients and what respondent (15) referred to as informal care givers. The introduction of e-health systems depends on people being willing to accept and use them, and the interviews provide examples of both acceptance and rejection. For staff, the main influence on acceptance is how they see a system affecting their work. For example, respondent (15) talked enthusiastically of a system that was useful and efficient – and was readily accepted:

It had a really positive impact in that information they'd never had before could, with a few clicks, bring a real, quick, easy clinical benefit. So that was a big thing where very quickly they could move information around quickly and safely. It improved communication and made things quicker.

Conversely, respondent (02) believed that systems fail if they take longer to deal with a task than it took without the software:

It will also fail if it is an add-on to a previous task. So if you write something in paper notes and then have to record it electronically, it's dead in the water. It will probably fail if it doesn't become part of the patient flow...Equally it must not be a barrier to the task, and that is quite a difficult balance to strike. Essentially the user needs to gain from it.

Several respondents commented that many health care staff see technology as intruding on their core task of caring for patients. Any administrative task is a distraction, whether paper or computer-based – so take an instinctively critical view of any system that appears to take longer than a manual system. This perception can apply even when the comparison between the two systems is wrong, since the e-health system may in fact be doing much more than the previous paper system. Respondent (08) believed that:

Clinicians particularly are very protectionist about their profession because technology opens the door to a range of people doing tasks that were traditionally done by others.

Other people themes included frustration amongst staff when systems they had asked for and believed to be necessary were delayed (01); the need to remember several passwords (01); the reaction of GPs to the possibility that they will be expected to share patient information with other professions in the care community, who have different views on data security (05); and the increasing ability of some patients to access medical information and use this to challenge their doctor's interpretation (06).

## Implementation processes

How people manage e-health projects – the implementation process – affects the degree to which they eventually become successfully integrated into routine health care services. The interviews examined the approaches to implementation used in e-health projects and how this helped or hindered implementation, under the headings of planning and adapting, participation, and power.

### Planning and adapting

Planning sets out in detail the steps needed to turn a policy decision into a working reality. It is not confined to the start of a project but continues iteratively throughout the task as people adapt plans to suit new and unforeseen conditions. In the simplest form it involves defining the tasks required to implement an e-health system, setting times by which they must be completed, and monitoring their progress – as a GP (03) leading a major project observed:

We had to work out who would be the right people to organise the electronic transfer and then we had to convince them that they should appoint a manager to run the project and prepare a business case and give it enough funding. And then they had to go to regional offices, set up pilot projects and consult with practice managers, user groups, practices, start in a very small way to see what the problems were and just gradually build up.

Setting out comprehensively what needs to be done only supports the outcome if people then complete them on time. Several respondents mentioned that getting e-health plans implemented took an excessive amount of time, using phrases such as 'it's just so slow', 'people seem to take for ever to do things', 'endless discussions in large committees that lead nowhere'. Respondent (01) provided a graphic example of the benefits of having an effective manager in charge:

It was great because she actually project managed it – if someone didn't do what they said they'd do by Tuesday, on the Wednesday morning they got a phone call to say 'this was meant to be done by yesterday, what's happening?' And people from the technology company said 'we've never had folk like you hassling us so much!' And the fact is that [too often] nobody actually gets off their backside and hassles folk.

### Participation

This refers to the extent to which those affected by an e-health project are involved in aspects of its planning, design and implementation. Most respondents focused on clinical participation in e-health projects, but some also mentioned policy makers and the Scottish Executive. Several mentioned the benefits of participation by support staff and 'informal care givers'. One (01) had chaired a user group:

There were probably two clinicians on when I was chair; rest were all e-health techie folk and that's why they wanted a clinical chair to keep them clinically driven. But it's still a very small clinical user base or clinical input. There's a lot of technical discussion and a lot of the time I would just say 'this is what we want to do, can you guys go and do it?' and they were happy with that...because they wanted the clinical input.

Discussing clinical-IT links respondent (13) explained that in their area:

the philosophy locally is to acknowledge the skills and expertise of IT colleagues and for clinicians and IT specialists to work together to find solutions, rather than have them in their own silos; so we've set up development communities where clinicians and IT developers work side by side to define requirements. We did that early on, they both have unique skill sets and if this is going to work they need to come together and get a common language [rather than IT specialists being remote from the clinical community].

Another had found users of the systems he had worked on welcomed them because the development team had taken great care to test upgrades rigorously, covering all possible eventualities, which usually led to a smooth implementation.

### Using power effectively

Power is the ability to influence others to act in a particular way, and as such its distribution and use affects the direction and nature of e-health provision. It affects the direction of e-health strategy, the resources devoted to it, the allocation of those resources to one project rather than another, and the balance between national and local applications.

Respondent (01) quoted a case in which the centre had withheld funding and so prevented further development work on a project which was about to be implemented, while (03) mentioned the failure to implement an electronic system for distributing lab results which was (and still is) a widely recognized problem. In both cases those promoting the projects had been unable to secure sufficiently powerful backing. Respondents (06, 12) gave examples of those in power obstructing e-health projects by supporting them in principle but then withholding funds; or under-estimating the resources which implementation required. Other political issues included the lack of sufficiently widespread clinical leadership with the power to advocate, and so gain acceptance (04, 05); and the importance of those wishing to implement a project being able to build their power by presenting a strong, credible case, and securing agreement at a sufficiently high level to ensure acceptance (03).

## Discussion

The interviews confirmed that many of the factors in the internal context which are known to affect information systems in general are also relevant to e-health projects. They also identified features of the implementation process which regularly appear in studies in other sectors. It is therefore imperative that those implementing e-Health services look to what has been learnt about implementation in other sectors and ensure that such knowledge is, where applicable, used to inform implementation in the health sector. Secondly, the practical significance of this is that it is clear that it is possible for those responsible for e-health to re-design the contextual factors to complement the e-health technology, in which case they will help people to accept and use the technology. Alternatively they can choose to design them in a way that discourages people from accepting and collaborating with the technology, in which case they hinder implementation.

## Conclusion

The results from this study imply that those responsible for managing e-health projects will be more successful if, amongst other things, they focus on managing five issues.

1. Embedding e-health applications in normal care activities, so that e-health clearly contributes to the wider health care strategy. They can aim to build the system into the normal patient workflow, and to adapt performance measurement systems to show how e-health applications support strategic targets.

2. Implementing national systems in a way that respects local conditions. To do this, senior managers need to set budgets that allow local managers to adapt the national system to local circumstances, and to meet the costs of training, maintenance and upgrades.

3. Designing systems that match, rather than challenge, the cultural values of a profession or unit. To do this, promoters need to identify the cultural values in the unit concerned, and work with people there to design a system that supports that culture, or alternatively to allow the time and resources needed to adapt the culture to the system.

4. Redesigning working processes in conjunction with e-health systems to support both policy and care needs. This involves taking time to understand current working practices as they actually are, and seeking users' advice on how e-health technologies and working practices can be jointly re-designed to improve performance.

5. Ensuring that people see systems as useful, by working with users to identify the information that is most useful to them in their tasks, and designing the e-health systems to provide that.

Above all, the interviews show that the scale of the e-health implementation task goes far beyond procuring the technology, since it involves redesigning the wider systems for delivering care. Those responsible for such projects can re-design the contextual factors to support the e-health technology, or they can design the e-health technology to fit the inherited context. Either course is likely to be more successful than concentrating on technology alone, without considering issues of context and process. In the next phase of our research we plan to examine how to transfer this knowledge into working practice.

## Competing interests

The authors declare that they have no competing interests.

## Authors' contributions

The authors listed on the title page contributed equally to this work

## Appendix 1 – Interview schedule

### Introduction – meaning of e-health

1. Can you tell me what the term e-Health means to you?

2. Can you describe your role in e-Health implementation and development?

3. How would you describe your general attitude towards technology?

Explain what we mean by e-Health – 'the application of information and communication technologies (ICT) across the whole range of functions that affect the health of citizens and patients'.

### Section 1 Current e-Health initiatives in place or in development

4. Having explained what we mean by e-Health, can you tell me what services or developments you are currently involved in?

5. Are you aware of anything being developed elsewhere?

Prompt

Management systems

Patient records

Transmission of data and images such as laboratory, x-rays or digital photos.

Booking systems

Communication systems

Email

Audio/Videoconferencing

Phone

Telepathology

Computerised decision support

Reminders or alerts for prescribing

Interactive, rule based decision making.

Information resources

Internet

Medical education

### Section 2 Factors that affect implementation and integration of e-Health

6. Can you choose one of these that we could talk about in more detail?

7. Can you talk me through what influenced how well that worked?

8. Research on the acceptance and use of technologies has identified factors which make affect whether the initiative will be successful and become part of normal practice. Can we discuss whether you think these factors were significant in the example we have just described?

9. How useful did you find it in your work? And how easy was it to use in practice?

10. Thinking about how much you enjoy your job and the satisfaction you get from your work, do you think this made any difference to this? If so how?

11. Do you think it had an impact on how the people in your organisation work together? Professional relationships?

12. Were there issues to do with accountability such as risk, security, confidentiality or safety when using the new systems?

13. Did it have an impact on the patient-professional interaction?

14. How has it impacted on the work of the organisation or its delivery of services? Performance, quality, budgets?

15. Can we take a little time to think about the culture you work in?

16. Would you describe it as entrepreneurial and visionary? Motivated by growth and creativity and supportive of its members. Or rational and efficiency seeking motivated by targets, suspicious of change. How did the example fit with that culture?

17. Can you tell me about the attitudes of patients towards it? Do you think they influenced how quickly and easily these initiatives were adopted?

18. How has the division of labour within the organisation been affected by the introduction of this? (Prompt: How has it affected who does what? Have you had to renegotiate the way people work (wages, rewards or status).

19. Do you think there were any issues about whether it was integrated as part of the existing provision or as a separate service?

20. Who would you say were most affected by it? Did you think any particular professional group facilitated or inhibited it's success?

21. Were there any financial considerations involved?

22. Were you aware of any patients or professionals being involved in the design? What sort of impact do think it had/would have had?

23. How much communication did you have about the implementation it? What sort of impact did that have?

24. In retrospect how effective has it been in doing what it was meant to do? How do you know this? Measured this?

25. What do you think have been the positive and negative outcomes of introducing it?

## Pre-publication history

The pre-publication history for this paper can be accessed here:

http://www.biomedcentral.com/1472-6947/9/9/prepub
